# Patient-reported improvements in health are maintained 2 years after completing a short course of cognitive behaviour therapy, exercise or both treatments for chronic widespread pain: long-term results from the MUSICIAN randomised controlled trial

**DOI:** 10.1136/rmdopen-2014-000026

**Published:** 2015-01-23

**Authors:** Marcus Beasley, Gordon J Prescott, Graham Scotland, John McBeth, Karina Lovell, Phil Keeley, Philip C Hannaford, Deborah P M Symmons, Ross I R MacDonald, Steve Woby, Gary J Macfarlane

**Affiliations:** 1 Musculoskeletal Research Collaboration (Epidemiology Group), Institute of Applied Health Sciences, University of Aberdeen, School of Medicine and Dentistry, Aberdeen, UK; 2 Medical Statistics Team, Institute of Applied Health Sciences, University of Aberdeen, School of Medicine and Dentistry, Aberdeen, UK; 3 Health Economic Research Unit, Institute of Applied Health Sciences, University of Aberdeen, School of Medicine and Dentistry, Aberdeen, UK; 4 Arthritis Research UK Centre of Excellence in Primary Care, Keele University, Keele, Staffordshire, UK; 5 School of Nursing, Midwifery and Social Work, The University of Manchester, Manchester, UK; 6 Centre for Academic Primary Care, Institute of Applied Health Sciences, University of Aberdeen, School of Medicine and Dentistry, Aberdeen, UK; 7 Arthritis Research UK Centre for Epidemiology, The University of Manchester, Manchester, UK; 8 Research and Development Department, The Pennine Acute Hospitals NHS Trust, North Manchester General Hospital, Manchester, UK

**Keywords:** Fibromyalgis/Pain Syndromes, Psychology, Economic Evaluations

## Abstract

**Objectives:**

The MUSICIAN study has previously shown short-term benefit but only marginal cost-effectiveness for two non-pharmacological interventions for chronic widespread pain (CWP). We wished to determine their long-term effectiveness and cost-effectiveness.

**Methods:**

A 2×2 factorial randomised controlled trial based in primary care in the UK. People were eligible if they were aged ≥25 years with CWP for which they had consulted their general practitioner. The interventions were a 6-month telephone cognitive behaviour therapy (tCBT) and/or a tailored exercise programme, in comparison to usual care. The primary outcome was patient-reported change in health.

**Results:**

884 persons were eligible, 442 were randomised and 81.7% were followed up 24 months post-treatment. In comparison to usual care (positive outcome 12.8%), tCBT (35.4%; OR 3.7 95% CI (1.8 to 8.0)), exercise (29.3%; OR 2.8 95% CI (1.3 to 6.0)) and both interventions (31.2%; OR 3.1 95% CI (1.3 to 6.0)) were significantly more effective. There was only a small decrease in effectiveness over time for individual and combined treatments. Those with more intense/disabling pain, higher distress and those who exhibited passive coping at baseline were more likely to have a positive outcome with tCBT than persons without these characteristics. tCBT was associated with the greatest increase in quality of life and lowest costs. Cost per quality adjusted life year was £3957–£5917 depending on method of analysis.

**Conclusions:**

A short course of tCBT for people with CWP was effective long-term and was highly cost-effective. Exercise was also effective but delivered positive outcome for fewer patients at greater cost, and there was no advantage for patients receiving both interventions.

**Trial registration number:**

ISRCTN67013851.

**Key messages**

- Telephone cognitive behaviour therapy (tCBT) and exercise have been shown in systematic reviews to be associated with short-term improvement of fibromyalgia symptoms, although the size of effects are modest.
- 6-month programmes of CBT delivered by telephone and exercise were associated with health improvement 2 years after the end of treatment, although there was no additional benefit of receiving both treatments.
- tCBT was highly cost-effective and improvement could partly be predicted by patient characteristics.

## Background

Chronic widespread pain (CWP: defined as axial skeleton pain and contra-lateral body pain present for at least 3 months) has a population prevalence of 11–14%.^1^ CWP is the cardinal feature of fibromyalgia, one of the most common reasons for referral to a rheumatologist.^2^ Longitudinal studies of CWP and fibromyalgia demonstrate that symptoms are persistent and long-lasting. In an 11-year follow-up of 1555 patients with fibromyalgia in the USA, substantial or moderate symptom improvement was observed in only 10% and 15% patients, respectively, while in 39%, symptoms worsened.^3^ In a follow-up of 173 adults with CWP in the UK only 15% were pain-free 7 years later.^4^

Although CWP symptoms are sometimes described as ‘unexplained’, epidemiological studies over the past two decades have provided important information on aetiology that has informed studies of management. Consistent findings in longitudinal population studies are that persons with poorer mental health (anxiety, depression and general psychological distress) and who take low levels of exercise have an increased risk of developing CWP or fibromyalgia.^5–7^ These risk factors offer targets for intervention: Bernardy *et al*^8^ concluded that cognitive behaviour therapy (CBT) resulted in increased coping with pain, reduced depressed mood and healthcare seeking behaviour; Haüser *et al*^9^ reported that an aerobic exercise programme resulted in decreased pain, and had positive effects on mood, health-related quality of life and physical fitness; and a network meta-analysis reported improved patient outcomes for both CBT and aerobic exercise.^10^ However, in a recent Cochrane review of CBT for fibromyalgia, the median duration of post-treatment follow-up for CBT interventions evaluated in trials was only 6 months post-treatment.^11^

We have previously reported the short-term (3 months post-treatment) results of the ‘Managing Unexplained Symptoms (chronic widespread pain) In primary Care: Involving traditional and Accessible New approaches’ (MUSICIAN) trial.^12^ These demonstrated significant clinical benefits of an individual or combined 6-month programme of CBT delivered by telephone (tCBT) and an exercise programme, compared with usual care. However, cost-effectiveness of the active interventions was marginal at 3 months post-treatment. In view of the positive clinical results we decided to conduct a long-term (24 months post-treatment), unplanned, follow-up to determine whether clinical benefits persisted, and to assess longer term cost-effectiveness. We also aimed to determine whether the characteristics of participants at trial entry predicted treatment response.

## Methods

A 2×2 factorial randomised controlled trial was conducted during 2008–2012. Trial participants, identified from the registered populations of eight general practices in Aberdeen, Scotland, and in Cheshire, England, were people aged ≥25 years who reported CWP according to the definition in the American College of Rheumatology (ACR) 1990 criteria for fibromyalgia,^13^ and for which they had consulted their general practitioner (GP) in the previous year. Exclusion criteria included contraindications to exercise, having pain that required specific alternative treatment or not having access to a landline telephone (for the delivery of CBT). Comorbid rheumatic disease was not an exclusion criterion. Participants were electronically randomised to treatment groups in blocks, stratified by pain intensity and disability (Chronic Pain Grade (CPG) questionnaire^14^) and psychological distress (General Health Questionnaire 12 item version (GHQ)^15^) A full description of those randomised into the trial has been reported previously.^12^

### Treatment groups

#### Telephone-delivered BCBT

This was delivered by therapists accredited by the British Association for Behaviour and Cognitive Psychotherapies who received 3 days of trial-specific training, a therapist manual and fortnightly clinical supervision. All sessions were digitally recorded for use in therapist supervision*.* Therapists mailed brief details welcoming patients to the study, giving a brief introduction to CBT and providing contact details. The intervention included an initial assessment (45–60 min), seven weekly sessions (each 30–45 min) delivered over 6 weeks, and a single session at 3 and 6 months postrandomisation. Therapists conducted a patient-centred assessment, developed shared understanding and formulation of the participants’ problem(s), and identified two to three patient-defined goals. Patients received a self-management CBT manual, ‘Managing Chronic Widespread Pain’, developed for the study (available from the authors). To enable patients to make an informed choice of the form of CBT they preferred, the manual included stories of fictitious patients using specific CBT techniques: behavioural activation (structured increasing of activities), cognitive restructuring (identifying and evaluating unhelpful thinking styles) and lifestyle changes (managing sleep, fatigue, irritability). Sessions involved implementing CBT techniques, working toward goals and problem solving barriers to improvement, while later sessions focused on relapse prevention.

#### Exercise

Experienced fitness instructors delivered the intervention and received a 1-day training session on exercise prescription for patients with CWP. They were observed during induction and follow-up meetings to monitor protocol adherence. Patients received a leisure-facility gym-based exercise programme consistent with American College of Sport Medicine (ACSM) guidelines for improving cardiorespiratory fitness.^16^ Following an induction session, patients were offered six fitness instructor-led monthly appointments for programme reassessment. Exercise intensity increased until levels were sufficient to achieve 40–85% of heart rate reserve. The ACSM does not prescribe specific exercises; these are negotiated between fitness instructor and patient. The trial protocol reflected this, allowing exercises to be changed while maintaining the goal of improving cardiorespiratory fitness. The exercise intensity range was broad, allowing individuals with low fitness or those who were deconditioned to achieve goals with low-intensity exercise. To avoid musculoskeletal injuries and to promote compliance, initial intensity was low to moderate. Patients were free to engage in additional exercises (eg, strength and flexibility training) to those prescribed. The recommended session duration was 20–60 min. Patients completed a diary recording frequency of gym attendance, exercise duration and type of exercise, which was returned to the coordinating unit. The ACSM guidelines recommend an exercise frequency of 3–5 days per week. This was thought to be unrealistic. Instead, patients were advised to attend at least twice a week, and on non-gym days engage in ‘everyday’ activities (eg, brisk walking) to enhance cardiorespiratory fitness.

#### Combined treatment

Participants randomised to this group received both of the above treatments concurrently.

#### Treatment as usual

Participants randomised to this group continued to receive usual care (without any restrictions) from their GP.

### Outcome measurements

Primary and secondary outcome data were collected at the end of treatment, and at 3 and 24 months post-treatment, by postal questionnaire. Non-responders were followed up with telephone interviews in which the primary outcome measure was recorded. The primary outcome measure was self-reported change in health status since the start of the trial; on a 7 point scale ranging from ‘Very much worse’ through ‘No change’ to ‘Very much better’. A positive outcome was defined as a report of ‘Much better’ or ‘Very much better’. This measure has been used previously in trials of exercise for fibromyalgia^17^ and for chronic fatigue syndrome.^18^

Secondary outcome measures were the Chalder Fatigue Scale,^19^ ^20^ pain (measured by the CPG), the Vanderbilt Pain Management Inventory,^21^ psychological distress (measured by the GHQ), the Sleep Problem Scale,^22^ the Tampa Scale for Kinesiophobia^23^ and the 36-Item Short Form Health Questionnaire (SF36).^24^

### Statistical issues: sample size and analysis

The sample size calculation in the registered protocol was based on change in the primary outcome measure at 3 months post-treatment. Anticipated improvements in the four arms (taking account of likely effectiveness and compliance with intervention) were: treatment as usual (TAU) 10%, exercise only 20%, CBT only 21.3%, exercise and CBT 31.3%. A total of 552 persons were deemed necessary to have at least 80% power of detecting differences in the active intervention groups compared with TAU. However, due to higher than anticipated follow-up rates during the trial, and the fact that the trial steering committee and data monitoring committee considered the original estimates (which used a χ^2^ test with continuity correction) to be too stringent, the trial sample size was reduced to 468.

Main treatment effects were assessed on an intention-to-treat analysis. The primary outcome was analysed using generalised estimating equations (GEE) for longitudinal logistic regression. A 2-way factorial regression using the outcome at end of treatment, 3 and 24 months post-treatment, including terms for treatment interaction and treatment–time effect, was fitted. The term for the interaction of treatments was less than one (ie, the combined effects were less than multiplicative).^12^ Analysis was carried out, therefore, comparing the three treatment groups to TAU. Secondary outcomes were analysed using GEE for longitudinal ordinal or linear regression where appropriate, including a treatment by time interaction with four separate treatment groups. Results are presented as ORs for logistic regression, proportional OR for ordinal regression and non-standardised regression coefficients for linear regression, with 95% CI for each active treatment compared with the TAU group at each time point, and for the treatment by time interaction. A Bonferroni correction allowed for multiple testing, with p values less than 0.004 considered statistically significant. Analyses were adjusted for age, sex, baseline CPG, baseline GHQ score and study centre, with analyses of secondary outcomes also adjusted for baseline scores on the outcome of interest. In order to determine the influence of missing follow-up data, we compared baseline data for participants who did and did not provide 24-month follow-up data. We also determined how sensitive the results were to missing follow-up data by, conservatively, assuming that all persons lost to follow-up data did not have a positive outcome on the primary measure, as well as performing analyses using imputation by chained equations, which predicts missing data based on all available data. All analyses were conducted using STATA software.^25^

To determine predictors of effectiveness of each intervention, logistic regression models were fitted separately for each outcome time point, to see which baseline factors (if any) modified treatment effectiveness. Age was treated as a continuous variable to calculate change in odds of treatment effectiveness for every 10 years. Other predictors were split into two categories by the median value. Four treatment groups were specified and models included an interaction between the baseline characteristic of interest and the treatment. ORs were calculated to compare the odds of improvement in each active treatment group to TAU. Then, a separate longitudinal model was fitted for each of the predictors of treatment effectiveness, to assess whether the effect was the same over all follow-up time points. Adjustment was made for the same baseline characteristics as in the main analysis.

### Health economic analysis

The UK national tariff^26^ was used to assign each participant with a health state utility weight based on their response to the EQ-5D at 24 months post-treatment. Reported health service resource use during the previous 6 months was valued using the same unit cost data used in the original analysis.^27–29^ Additional quality adjusted life years (QALYs) accrued between 3 and 24 months post-treatment were calculated for each participant assuming a linear change in utility. This was added to the 3-month post-treatment QALY estimate for each patient. Linear interpolation between reported health service costs at 3 and 24 months post-treatment was used to impute an average quarterly cost for each patient for each of the five quarters not covered by data collection.^30^ Costs and QALYs incurred beyond 12 months were discounted at the rate of 3.5% per annum in line with accepted practice in the UK.

Multivariate regression analysis estimated differences in mean costs and QALYs between the three active treatment groups and TAU. A generalised linear model, with a γ family distribution and a log link function, was specified to account for the skewed nature of the cost data. Cost-effectiveness acceptability curves were constructed using non-parametric bootstrapping and the net monetary benefit framework, to determine the probability of the alternative interventions being considered cost-effective at different ceiling ratios representing society's willingness to pay (WTP) per QALY (£20 000–£30 000 per QALY are commonly applied ceiling ratios in the UK). The analysis was initially conducted for participants with complete cost and QALY data at final follow-up. Multiple imputation analyses, using chained equations, were used to assess the sensitivity of findings to missing data.

## Results

**Figure 1:**
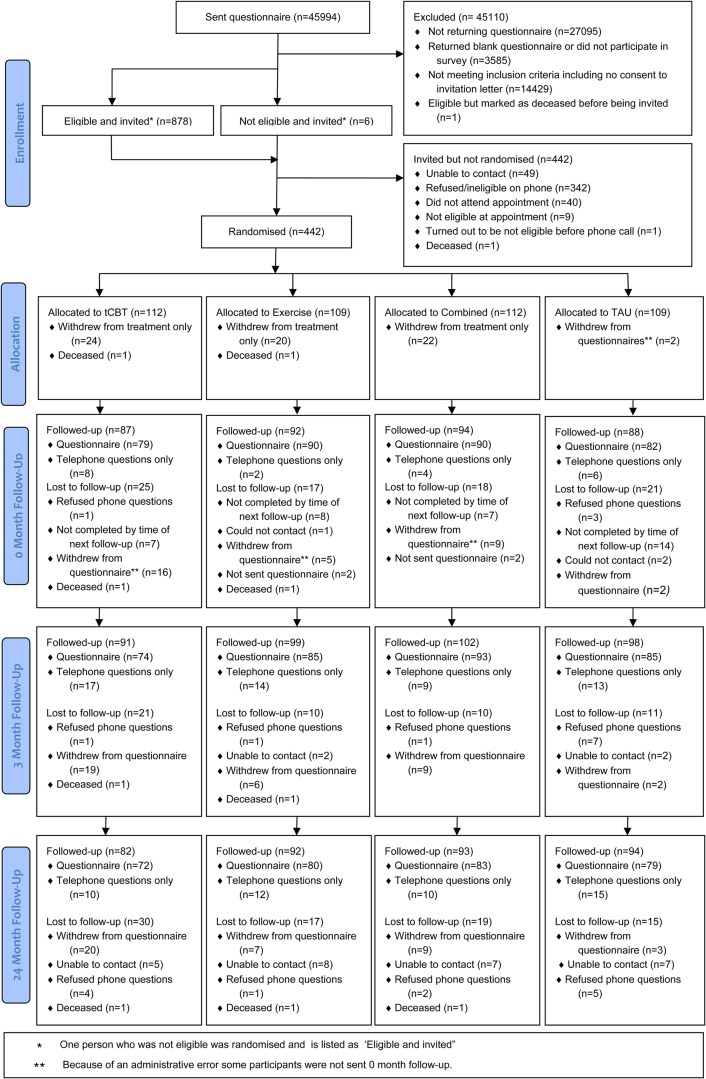
MUSICIAN Trial CONSORT flow diagram (TAU, treatment as usual; tCBT, telephone cognitive behavioural therapy).

In total, 884 people were identified as eligible and invited to participate in the trial, and 442 (50%) were randomised (figure 1). Those randomised had a mean age of 56.2 years (range 25–85 years), 69.5% were women and 33.9% were in full-time employment. The CWP was graded as CPG III-IV for 30% of participants. In comparison to all those identified as eligible, those randomised were more likely to be older, have a higher body mass index and have more severe pain (p<0.05), with no other differences found (table 1). There was no important or statistically significant difference in any of the secondary outcome measures across treatment groups.^12^

**Table 1:** Baseline characteristics of participants (n=442)

Age*, years	56.3 (13.0)
Gender, female	307 (69.5)
Employment status	
Working full-time	154 (34.8)
Working part-time	68 (15.4)
Retired	142 (32.1)
Other	78 (17.6)
Chronic Pain Grade	
I	92 (20.8)
II	174 (39.4)
III	97 (22.0)
IV	79 (17.9)
GHQ*	3.2 (3.6)
EQ-5D*	0.69 (0.19)
SF-36*	
Physical component	40.6 (7.9)
Mental component	45.2 (10.7)
Fatigue*	19.6 (5.9)
VPMI*	
Passive coping	29.5 (7.3)
Active coping	24.9 (4.2)
Sleep scale*	9.2 (5.6)
Tampa Scale for Kinesiophobia*	35.8 (5.3)

Values are n (%) except * which are mean (SD).

GHQ, General Health Questionnaire; SF-36, 36-item Short Form Health Questionnaire; VPMI, Vanderbilt Pain Management Inventory.

### Primary outcome

At 24 months post-treatment, 361 participants were followed up (81.7%). Of these, 12.8% in the TAU group reported a positive outcome compared with 35.4% in the tCBT group, 29.3% in the exercise group and 31.2% in the combined treatment group (table 2). The adjusted OR for reporting a positive outcome compared with the TAU group were tCBT OR 3.6 (95% CI 1.7 to 7.6), exercise 2.5 (95% CI 1.2 to 5.4) and combined treatment 2.9 (95% CI 1.4 to 6.0).

**Table 2:** Primary and secondary outcomes at 24 months post-treatment

	TAU N=94	tCBT N=82	Exercise N=92	Combined N=93
*Primary outcome: global change in health since entering trial*				
Original data				
Very much better	3 (3.2)	5 (6.1)	5 (5.4)	10 (10.8)
Much better	9 (9.6)	24 (29.3)	22 (23.9)	19 (20.4)
A little better	14 (14.9)	15 (18.3)	16 (17.4)	17 (18.3)
No change	31 (33.0)	21 (25.6)	22 (23.9)	19 (20.4)
A little worse	21 (22.3)	9 (11.0)	16 (17.4)	18 (19.4)
Much worse	7 (7.5)	8 (9.8)	9 (9.8)	10 (10.8)
Very much worse	9 (9.6)	0	2 (2.2)	0
Dichotomised outcome				
Much better/very much better	12 (12.8)	29 (35.4)	27 (29.3)	29 (31.2)
Less than much better	82 (87.2)	53 (64.6)	65 (70.7)	64 (68.8)
*Secondary outcomes*				
CPG				
0—No pain	14 (19.4)	12 (18.2)	10 (14.1)	10 (13.5)
I—Low disability, low intensity	18 (25.0)	22 (33.3)	23 (32.4)	29 (39.2)
II—Low disability, high intensity	22 (30.6)	20 (30.3)	16 (22.5)	21 (28.4)
III—High disability, moderately limiting	11 (15.3)	8 (12.1)	15 (21.1)	8 (10.8)
IV—High disability, severely limiting	7 (9.7)	4 (6.1)	7 (9.9)	6 (8.1)
GHQ*	3.0 (3.3)	2.2 (3.4)	2.6 (3.7)	3.0 (3.7)
EQ-5D*	0.63 (0.32)	0.73 (0.24)	0.71 (0.24)	0.68 (0.24)
SF-36* (all 1–100)†				
General health	56.2 (21.3)	60.4 (20.1)	59.9 (22.3)	61.5 (23.3)
Physical function	59.2 (31.9)	66.9 (26.8)	68.9 (25.9)	68.3 (24.8)
Role physical	59.7 (30.7)	63.3 (27.0)	62.9 (25.2)	68.4 (29.3)
Vitality	45.0 (22.1)	50.6 (21.9)	49.8 (20.0)	49.2 (22.8)
Social function	68.5 (28.2)	74.8 (26.3)	71.4 (26.1)	76.2 (25.5)
Role emotional	73.7 (27.3)	76.5 (26.4)	79.7 (23.2)	74.1 (29.3)
Fatigue (0–42)*	19.1 (7.3)	17.8 (6.6)	17.8 (5.8)	17.6 (6.9)
VPMI*				
Passive coping (11–55)	29.4 (8.9)	25.8 (8.4)	27.5 (8.8)	25.3 (8.2)
Active coping (7–35)	24.5 (4.6)	25.2 (4.2)	25.1 (3.3)	25.8 (4.4)
Sleep scale*(0–20)	9.7 (5.9)	9.4 (6.0)	8.8 (5.2)	8.3 (6.0)
Tampa Scale for Kinesiophobia*(17–68)	36.2 (7.2)	33.3 (5.7)	34.1 (7.6)	32.9 (7.8)

Values are n (%) except * which are mean (SD).

†Bodily Pain scores not available due to printing error on questionnaire.

CPG, Chronic Pain Grade questionnaire; GHQ, General Health Questionnaire; SF-36, 36 item Short Form Health Questionnaire; TAU, treatment as usual; tCBT, telephone cognitive behavioural therapy; VPMI, Vanderbilt Pain Management Inventory.

Each treatment group was associated with statistically significant increased odds of a positive outcome at each time point compared with TAU (table 3). The odds of reporting a positive outcome showed a small decrease with time for all active treatments (change in OR/month 0.96 to 0.99).

**Table 3:** Estimations of treatment effectiveness at end of treatment, 3 and 24 months post-treatment calculated from longitudinal model (adjusted ORs*, 95% CIs and p values)

	End of treatment	3 months post-treatment	24 months post-treatment	Change in OR/month
tCBT	5.0	4.8	3.6	0.99
	(2.4 to 10.6)	(2.4 to 9.7)	(1.7 to 7.6)	(0.95 to 1.02)
	<0.001	<0.001	0.001	0.410
Exercise	4.6	4.3	2.5	0.98
	(2.2 to 9.6)	(2.1 to 8.5)	(1.2 to 5.4)	(0.94 to 1.01)
	<0.001	<0.001	0.015	0.147
Combined treatment	6.7	6.0	2.9	0.96
	(3.3 to 13.9)	(3.1 to 11.9)	(1.4 to 6.0)	(0.93 to 0.997)
	<0.001	<0.001	0.006	0.035

*Results of the four-group longitudinal logistic regression model, adjusting for age, sex, centre, and baseline CPG and GHQ scores, including a term for treatment–time interaction.

CPG, Chronic Pain Grade questionnaire; GHQ, General Health Questionnaire; tCBT, telephone cognitive behavioural therapy.

### Secondary outcomes

The active treatment groups were generally associated with small improvements in each of the secondary measures compared with TAU (tables 4 and 5), but these tended to decrease over time. At 24 months, participants in the combined treatment group (in comparison to the TAU group) had significantly (p<0.004) reduced passive coping, kinesiophobia and improved SF-36 role physical; with significant improvement in four other SF-36 subscales that did not persist after correction for multiple testing. The tCBT group showed significant improvement at 24 months (in comparison to the TAU group) in passive coping, kinesiophobia, distress and SF-36 social function subscale but these did not persist after correction for multiple testing. The exercise group showed (in comparison to the TAU group) a significant improvement in SF-36 role emotional at 24 months that did not persist after correction for multiple testing.

**Table 4:** Treatment effectiveness, secondary outcomes (values are unstandardised coefficients except CPG, which are proportional ORs)*

	End of treatment	3 months post-treatment	24 months post-treatment	Treatment–time interaction (slope)
CPG				
tCBT	0.4†	0.5†	0.7	1.02
	(0.3 to 0.8)	(0.3 to 0.8)	(0.3 to 1.3)	(0.99 to 1.05)
Exercise	0.7	0.8	1.3	1.02
	(0.5 to 1.1)	(0.5 to 1.2)	(0.7 to 2.4)	(0.99 to 1.05)
Combined treatment	0.5†	0.5‡	0.9	1.03
	(0.3 to 0.8)	(0.3 to 0.8)	(0.4 to 1.8)	(1.00 to 1.06)
GHQ				
tCBT	−1.0‡	−1.0‡	−1.0‡	0.00
	(−1.8 to −0.3)	(−1.8 to −0.3)	(−1.9 to −0.1)	(−0.03 to 0.04)
Exercise	−0.9‡	−0.9‡	−0.5	0.02
	(−1.7 to −0.2)	(−1.6 to −0.2)	(−1.4 to 0.3)	(−0.02 to 0.05)
Combined treatment	−1.0‡	−0.8‡	0.0	0.04‡
	(−1.7 to −0.2)	(−1.5 to −0.1)	(−0.9 to 0.9)	(0.00 to 0.08)
Fatigue				
tCBT	−2.6‡	−2.4‡	−1.0	0.06
	(−4.4 to −0.7)	(−4.1 to −0.6)	(−3.0 to 1.1)	(−0.01 to 0.14)
Exercise	−2.4‡	−2.2‡	−1.2	0.05
	(−4.1 to −0.7)	(−3.9 to −0.6)	(−3.2 to 0.8)	(−0.02 to 0.12)
Combined treatment	−4.4†	−4.0†	−1.5	0.12†
	(−6.1 to −2.6)	(−5.7 to −2.3)	(−3.5 to 0.5)	(0.04 to 0.20)
VPMI passive coping				
tCBT	−2.0‡	−2.0‡	−2.7‡	−0.03
	(−3.7 to −0.2)	(−3.7 to −0.4)	(−4.7 to −0.7)	(−0.10 to 0.04)
Exercise	−2.0‡	−2.0‡	−1.3	0.03
	(−3.6 to −0.3)	(−3.5 to −0.3)	(−3.2 to 0.6)	(−0.04 to 0.10)
Combined treatment	−3.1†	−3.1†	−3.1†	0.00
	(−4.7 to −1.5)	(−4.7 to −1.5)	(−5.0 to −1.2)	(−0.07 to 0.07)
VPMI active coping				
tCBT	0.8	0.8	0.8	0.00
	(−0.1 to 1.7)	(0.0 to 1.6)	(−0.3 to 1.8)	(−0.05 to 0.04)
Exercise	1.1‡	1.1‡	0.7	−0.02
	(0.3 to 2.0)	(0.3 to 1.9)	(−0.3 to 1.8)	(−0.06 to 0.03)
Combined treatment	1.3†	1.3†	0.8	−0.02
	(0.5 to 2.1)	(0.5 to 2.0)	(−0.2 to 1.8)	(−0.07 to 0.02)
Sleep problems				
tCBT	−1.8‡	−1.7‡	−0.8	0.04
	(−3.0 to −0.6)	(−2.8 to −0.5)	(−2.3 to 0.6)	(−0.02 to 0.10)
Exercise	−1.2‡	−1.2‡	−0.9	0.01
	(−2.4 to −0.1)	(−2.3 to −0.1)	(−2.3 to 0.5)	(−0.04 to 0.07)
Combined treatment	−1.5‡	−1.4‡	−0.8	0.03
	(−2.6 to −0.3)	(−2.5 to −0.2)	(−2.2 to 0.6)	(−0.03 to 0.08)
TSK				
tCBT	−1.4	−1.5	−1.9‡	−0.02
	(−3.0 to 0.1)	(−3.0 to 0.0)	(−3.6 to −0.2)	(−0.08 to 0.04)
Exercise	−1.5	−1.4	−1.2	0.01
	(−3.0 to 0.0)	(−2.9 to 0.0)	(−2.9 to 0.5)	(−0.04 to 0.07)
Combined treatment	−2.4†	−2.5†	−2.6†	−0.01
	(−3.9 to −1.0)	(−3.9 to −1.0)	(−4.3 to −0.9)	(−0.06 to 0.05)

*Comparison group—treatment as usual. All models are adjusted for age, sex, centre, baseline CPG and GHQ scores, and baseline levels of outcome of interest.

†p<0.004, with correction for multiple testing.

‡p<0.05.

CPG, Chronic Pain Grade questionnaire; GHQ, General Health Questionnaire; TAU, treatment as usual; tCBT, telephone cognitive behavioural therapy; TSK, Tampa Scale for Kinesiophobia; VPMI, Vanderbilt Pain Management Inventory.

**Table 5:** Treatment effectiveness, secondary outcomes (SF-36*) (values are unstandardised coefficients)†

	End of treatment	3 months post-treatment	24 months post-treatment	Treatment–time interaction (slope)
SF36—general health				
tCBT	2.4	2.7	4.4	0.08
	(−1.7 to 6.5)	(−1.3 to 6.6)	(−0.2 to 9.1)	(−0.09 to 0.26)
Exercise	2.5	2.7	4.0	0.06
	(−1.5 to 6.4)	(−1.1 to 6.5)	(−0.6 to 8.5)	(−0.11 to 0.23)
** **Combined treatment	5.1‡	5.1‡	5.1‡	0.00
	(1.2 to 9.0)	(1.3 to 8.9)	(0.6 to 9.6)	(−0.17 to 0.17)
SF36—physical function				
** **tCBT	3.5	3.5	3.3	−0.01
	(−0.9 to 7.9)	(−0.8 to 7.7)	(−1.7 to 8.3)	(−0.19 to 0.17)
** **Exercise	6.4§	6.2§	4.7	−0.07
	(2.2 to 10.6)	(2.1 to 10.3)	(−0.1 to 9.5)	(−0.24 to 0.10)
** **Combined treatment	8.8§	8.5§	6.2‡	−0.11
	(4.6 to 13.0)	(4.4 to 12.5)	(1.4 to 10.9)	(−0.28 to 0.06)
SF36—role physical				
** **tCBT	5.8‡	5.7‡	4.6	−0.05
	(0.2 to 11.5)	(0.3 to 11.1)	(−2.0 to 11.2)	(−0.31 to 0.21)
** **Exercise	4.4	4.3	3.5	−0.04
	(−1.1 to 9.8)	(−0.9 to 9.5)	(−2.9 to 9.9)	(−0.29 to 0.22)
** **Combined treatment	7.7‡	8.0§	9.8§	0.08
	(2.3 to 13.1)	(2.8 to 13.1)	(3.4 to 16.1)	(−0.17 to 0.34)
SF36—vitality				
** **tCBT	5.4‡	5.4‡	5.1	−0.01
	(0.8 to 10.1)	(0.9 to 9.8)	(−0.3 to 10.5)	(−0.22 to 0.19)
** **Exercise	2.6	2.7	4.0	0.06
	(−1.9 to 7.0)	(−1.5 to 7.0)	(−1.2 to 9.3)	(−0.14 to 0.26)
** **Combined treatment	5.0‡	5.1‡	5.5‡	0.02
	(0.6 to 9.4)	(0.8 to 9.4)	(0.3 to 10.7)	(−0.18 to 0.22)
SF36—social function				
** **tCBT	7.4‡	7.4‡	7.3‡	−0.01
	(1.9 to 12.9)	(2.2 to 12.6)	(0.8 to 13.7)	(−0.27 to 0.26)
** **Exercise	7.7‡	7.0‡	2.5	−0.22
	(2.4 to 12.9)	(2.0 to 12.0)	(−3.8 to 8.7)	(−0.47 to 0.04)
** **Combined treatment	6.7‡	6.8‡	7.8‡	0.05
	(1.4 to 11.9)	(1.8 to 11.8)	(1.6 to 14.0)	(−0.21 to 0.30)
SF36—role emotional				
** **tCBT	8.3§	7.7§	3.2	−0.21
	(2.8 to 13.8)	(2.5 to 12.8)	(−3.4 to 9.7)	(−0.49 to 0.07)
** **Exercise	9.1§	8.8§	6.4‡	−0.11
	(3.9 to 14.4)	(3.8 to 13.7)	(0.1 to 12.8)	(−0.38 to 0.16)
** **Combined treatment	8.0§	7.1‡	1.0	−0.29‡
	(2.8 to 13.2)	(2.2 to 12.0)	(−5.3 to 7.4)	(−0.56 to 0.02)

*The combined scales of the SF-36 are not available due to a printing error in the follow-up questionnaire.

†Comparison group is treatment as usual. Values are unstandardised coefficients with 95% CIs. All models are adjusted for age, sex, centre, baseline CPG and GHQ scores, and baseline levels of outcome of interest.

‡p<0.05.

§p<0.004, with correction for multiple testing.

CPG, Chronic Pain Grade questionnaire; GHQ, General Health Questionnaire; tCBT, telephone cognitive behavioural therapy; SF-36, 36-item Short Form Health Questionnaire.

### Influence of missing data

Comparing the baseline data of responders (n=361) and non-responders (n=81) at 24 months post-treatment, the latter were more likely to have had CPG IV (15.5% and 28.4%, respectively) but there were no other statistically significant, sizeable or clinically important differences in demographic or clinical variables assessed. Assuming, conservatively, that all participants who did not provide outcome data at 24 months did not have a positive primary outcome, the percentage of participants with a positive outcome across the four groups was tCBT 25.9%, exercise 24.8%, combined intervention 25.9%, TAU 11%. Differences between the intervention groups and TAU remained statistically significant (OR for positive outcome compared with usual care: tCBT (OR 2.8 (95% CI 1.4 to 5.9), exercise 2.7 (95% CI 1.3 to 5.6), combined 2.8 (95% CI 1.4 to 5.9)). Imputation produced results that were very similar to those reported in table 2, with any small differences not affecting the interpretation of findings (data not shown).

### Predictors of treatment effectiveness

Potential predictors of treatment effectiveness are shown in table 6. Participants with more intense or disabling pain (as measured by CPG) or higher levels of distress (measured by GHQ) benefitted more from tCBT or combined treatment (compared to those without these characteristics) and participants with higher levels of kinesiophobia were more likely to benefit from tCBT.

**Table 6:** Predictors of treatment effectiveness

	Months post-treatment			Longitudinal adjusted model
	0*	3	24	
*Gender*				
Male vs female				
TAU	1	1	1	1
tCBT	0.5 (0.1 to 3.9)	1.6 (0.2 to 11.5)	0.2 (0.0 to 1.5)	0.4 (0.1 to 2.1)
Exercise	0.6 (0.1 to 4.2)	0.8 (0.1 to 5.6)	0.2 (0.0 to 2.1)	0.4 (0.1 to 1.7)
Combined	0.5 (0.1 to 3.7)	0.8 (0.1 to 5.4)	0.2 (0.0 to 2.3)	0.4 (0.1 to 1.8)
*CPG*				
3/4 vs 1/2				
TAU	1	1	1	1
tCBT	1.2 (0.2 to 8.4)	3.5 (0.3 to 35.4)	*11.8 (1.2 to 115.8)*	4.8 (0.9 to 25.6)
Exercise	0.9 (0.1 to 6.2)	2.9 (0.3 to 30.0)	5.3 (0.5 to 52.5)	3.4 (0.6 to 17.9)
Combined	2.3 (0.0 to 15.1)	*10.6 (1.1 to 105.6)*	8.9 (0.9 to 86.8)	*8.3 (1.6 to 43.4)*
*Age*				
Per 10 years				
TAU	1	1	1	1
tCBT	1.3 (0.6 to 2.8)	1.1 (0.5 to 2.1)	1.5 (0.8 to 2.7)	1.2 (0.7 to 2.0)
Exercise	*2.2 (1.1 to 4.6)*	1.3 (0.7 to 2.7)	0.9 (0.5 to 1.7)	1.4 (0.8 to 2.2)
Combined	*2.5 (1.2 to 5.1)*	1.5 (0.8 to 3.0)	1.2 (0.7 to 2.2)	1.6 (0.98 to 2.6)
*GHQ12*				
3–12 vs 0–2				
TAU	1	1	1	1
tCBT	3.4 (0.5 to 20.8)	4.0 (0.7 to 23.0)	*7.7 (1.5 to 40.7)*	*5.6 (1.5 to 21.2)*
Exercise	0.8 (0.1 to 4.6)	1.7 (0.3 to 10.0)	4.5 (0.9 to 23.6)	2.3 (0.6 to 8.5)
Combined	1.5 (0.2 to 8.5)	1.5 (0.3 to 8.4)	*6.7 (1.3 to 34.7)*	3.2 (0.9 to 11.9)
*Fatigue*				
19–40 vs 8–18				
TAU	1	1	1	1
tCBT	0.6 (0.1 to 4.0)	0.7 (0.1 to 3.7)	1.7 (0.4 to 8.2)	1.0 (0.3 to 3.8)
Exercise	0.2 (0.0 to 1.7)	0.4 (0.1 to 2.6)	0.8 (0.2 to 4.0)	0.6 (0.2 to 2.1)
Combined	1.1 (0.2 to 7.8)	1.4 (0.3 to 8.0)	4.5 (0.9 to 21.8)	2.7 (0.7 to 9.6)
*Passive coping*				
30–52 vs 18–29				
TAU	1	1	1	1
tCBT	1.5 (0.2 to 10.3)	2.2 (0.4 to 12.8)	2.1 (0.4 to 10.4)	1.6 (0.4 to 5.7)
Exercise	1.0 (0.1 to 7.0)	0.5 (0.1 to 2.9)	1.1 (0.2 to 5.1)	0.8 (0.2 to 2.9)
Combined	3.7 (0.6 to 24.7)	2.2 (0.4 to 11.9)	1.6 (0.3 to 7.5)	2.1 (0.6 to 7.5)
*Active coping*				
26–35 vs 10–25				
TAU	1	1	1	1
tCBT	0.9 (0.1 to 5.5)	0.6 (0.1 to 3.5)	1.6 (0.3 to 7.1)	1.0 (0.3 to 3.4)
Exercise	2.2 (0.4 to 13.5)	1.5 (0.3 to 8.7)	1.7 (0.4 to 7.5)	1.7 (0.5 to 6.1)
Combined	0.7 (0.1 to 4.2)	0.6 (0.1 to 3.2)	0.3 (0.1 to 1.5)	0.7 (0.2 to 2.4)
*Sleep problems*				
10–20 vs 0–9				
TAU	1	1	1	1
tCBT	1.5 (0.2 to 9.3)	1.0 (0.2 to 5.9)	2.9 (0.6 to 14.1)	1.8 (0.5 to 6.5)
Exercise	1.9 (0.3 to 11.1)	2.1 (0.4 to 12.2)	2.5 (0.5 to 11.8)	2.4 (0.7 to 8.5)
Combined	1.2 (0.2 to 7.2)	0.9 (0.2 to 5.0)	1.0 (0.2 to 5.2)	1.1 (0.3 to 3.9)
*TSK*				
37–57 vs 21–36				
TAU	1	1	1	1
tCBT	2.0 (0.3 to 13.8)	3.7 (0.6 to 24.3)	*10.6 (1.7 to 66.3)*	*4.7 (1.1 to 20.5)*
Exercise	2.4 (0.4 to 16.3)	2.6 (0.4 to 17.3)	1.8 (0.3 to 11.8)	2.8 (0.7 to 12.1)
Combined	3.9 (0.6 to 26.6)	2.6 (0.4 to 16.6)	3.7 (0.6 to 23.1)	3.6 (0.8 to 15.1)

*ORs with 95% CIs. Italic typeface indicates statistical significance.

CPG, Chronic Pain Grade questionnaire; GHQ, General Health Questionnaire; TAU, treatment as usual; tCBT, telephone cognitive behavioural therapy; TSK, Tampa Scale for Kinesiophobia.

### Health economics

**Figure 2:**
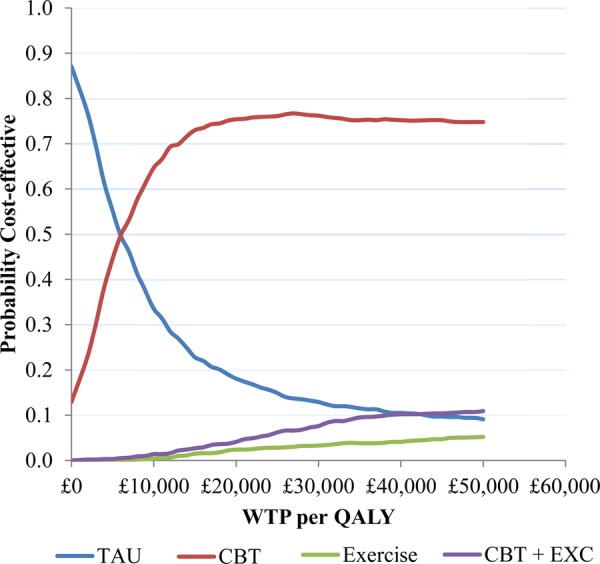
Cost-effectiveness acceptability curves using generalised linear model with γ distribution and log link function to estimate incremental costs and QALYs (complete case data). CBT, cognitive behavioural therapy; QALY, quality adjusted life year; TAU, treatment as usual; WTP, willingness to pay; EXC, exercise.

Treatment costs during the intervention period, and post-treatment follow-up costs, are summarised in table 7. The cost-effectiveness analysis showed that all of the active treatments were associated with an increased cost to the health service and an increase in QALYs compared with TAU (table 8). tCBT was associated with the lowest cost increase and highest QALY gain, and is therefore dominant over the alternative active treatments. Based on analysis of persons who provided complete data, the additional cost per QALY gained with tCBT versus TAU was £5917. Based on the results of the non-parametric bootstrap, tCBT was found to have an approximately 75% chance of being the preferred strategy at a ceiling ratio of £20 000 per QALY gained (figure 2). The general pattern of results remained the same with multiple imputation for missing data, although the additional cost per QALY gained for tCBT reduced to £3957.

**Table 7:** Estimated resource use, costs and utilities by treatment allocation group at 24 months post-treatment

	TAU (N=109)			CBT (N=112)			Exercise (N=109)			CBT+exercise (N=112)		
	N	Mean	SD	N	Mean	SD	N	Mean	SD	N	Mean	SD
Intervention costs 0–6 months*	109	0	0	112	205	136	109	456	126	112	698	190
Routine health service costs to 3 months post-treatment*	67	837	1808	66	819	2112	76	807	1318	83	803	1320
Routine health service resource use (18–24 months)												
GP visits	79	3.19	4.76	72	2.32	1.92	80	2.70	2.24	84	2.70	2.60
Practice nurse visits	79	1.22	2.21	72	1.76	5.72	80	1.16	1.91	84	1.13	1.88
Community physio visits	79	0.65	2.37	72	0.54	2.11	80	0.40	1.73	84	0.79	2.30
Other community visits	79	0.43	1.77	72	1.00	3.91	80	0.24	0.82	84	0.63	1.97
Outpatient visits	79	0.84	1.18	72	1.03	1.43	80	0.75	1.31	84	1.00	1.57
Hospital physio and other services	78	1.69	5.08	72	0.64	1.97	80	1.36	4.09	84	0.93	2.96
Inpatients’ admission days	79	0.29	1.24	72	0.30	0.94	80	0.71	1.99	84	0.76	2.26
Health service costs (18–24 months)												
Primary care costs (18–24 months post-treatment)	79	£140	£185	72	£115	£124	80	£115	£97	84	£126	£135
Outpatient costs (18–24 months)	79	£99	£138	72	£120	£167	80	£90	£154	84	£117	£183
Hospital physio and other services (18–24 months post-treatment)	79	£97	£332	72	£39	£127	80	£80	£221	84	£82	£316
Hospital admissions costs (18–24 months post-treatment)	79	£181	£671	72	£254	£694	80	£452	£1184	84	£441	£1138
Total health service costs (18–24 months)	79	£516	£911	72	£529	£832	80	£737	£1382	84	£764	£1466
Imputed quarterly cost (3–18 months)	68	£206	£316	64	£248	£339	72	£317	£433	80	£308	£437
Total NHS costs (randomination—24 months post-treatment)	59	£2387	£3885	60	£2925	£3962	65	£3616	£3849	72	£3715	£4078
Utilities												
EQ-5D baseline	108	0.649	0.216	112	0.730	0.151	108	0.686	0.209	111	0.681	0.175
EQ-5D 6 months	81	0.688	0.245	76	0.723	0.266	89	0.716	0.208	86	0.737	0.176
EQ-5D 9 months	83	0.645	0.262	71	0.754	0.214	81	0.705	0.238	90	0.701	0.220
QALYs (randomisation—3 months post-treatment)	67	0.516	0.144	64	0.548	0.141	71	0.539	0.122	78	0.537	0.110
EQ-5D (24 months post-treatment)	78	0.631	0.315	70	0.730	0.242	78	0.712	0.242	79	0.682	0.238
QALYs (randomisation—24 months post-treatment)	61	1.697	0.543	56	1.825	0.474	61	1.798	0.490	65	1.752	0.440

*Detailed breakdown of costs previously reported.

CBT, cognitive behavioural therapy; GP, general practitioner; NHS, National Health Service; QALY, quality adjusted life year; TAU, treatment as usual.

**Table 8:** Adjusted incremental costs and QALYs for the active treatments versus treatment as usual (using complete cases and multiple imputation data at 24 months post-treatment)

	Incremental cost mean (95% CI)	Incremental QALYs; mean (95% CI)	Additional cost per QALY*
*Complete cases*			
TAU (n=59)	†	†	†
tCBT (n=53)	£574 (−£441 to £1554)	0.097 (−0.048 to 0.240)	£5917
Exercise (n=61)	£1924 (£782 to £3295)	0.025 (−0.099 to 0.154)	Dominated
Combined (n=61)	£1778 (£690 to £3009)	0.047 (−0.086 to 0.182)	Dominated
*Multiple imputation data*			
TAU	†	†	†
tCBT	£554 (−£121 to £1297)	0.140 (0.046 to 0.236)	£3957
Exercise	£1256 (£488 to £2097)	0.071 (−0.019 to 0.165)	Dominated
Combined	£1453 (£684 to £2300)	0.096 (0.000 to 0.189)	Dominated

*Incremental cost-effectiveness ratio (ICER) expressed relative to next less costly non-dominated alternative. †Reference group.

QALY, quality adjusted life year; TAU, treatment as usual; tCBT, telephone cognitive behavioural therapy.

## Discussion

We have shown that after a short-course of tCBT and/or a personalised exercise programme, approximately one-third of patients with CWP reported a positive primary outcome (change in condition) 24 months after end of treatment, significantly better than patients receiving TAU, where the improvement was one in eight. tCBT and exercise appeared to be similarly beneficial, and there was no advantage gained from providing both. Combined treatment, however, did appear to produce greater improvements in several secondary outcome measures 24 months post-treatment (compared to usual care and after correction for multiple testing). tCBT was highly cost-effective in the long-term, with the cost per QALY ranging between approximately £4k and £6k, depending on the method of analysis.

A number of points should be considered when interpreting our results. First, participants reported CWP (rather than having a diagnosis of fibromyalgia) and were recruited through primary care. Thus, many in the study population had symptoms that were less severe, as evidenced by the CPG and reported work status, than would typically be seen by rheumatologists. Second, it could be argued that the positive results were due to non-specific benefits from participating in a trial rather than the specific effects of the interventions delivered. Supporting such an interpretation is the similarity of positive effects across all active intervention groups (including the group receiving both interventions). Against this interpretation, CWP has proved very difficult for rheumatologists and others to treat, and so it seems unlikely that such strongly positive improvements resulted from simply ‘attention’. We also demonstrated improvements in some of the secondary outcomes related to participants’ perceptions of improvement in their condition. If our initial results were due to non-specific effects, we would expect such effects to wane with time. Instead, we have observed persistence of strong effects over 2 years. Furthermore, we have demonstrated that persons more likely to benefit from tCBT have characteristics that tCBT seeks to change. We recognise, however, that not all patients with CWP will necessarily be willing to consider undertaking exercise or participating in a CBT programme. Our results, therefore, can only be extrapolated to those willing to do so and, in this study, 70% of persons randomised to tCBT completed at least six sessions while 50% of persons randomised to exercise attended the gym at least two times per week. Third, the study was not powered to undertake a robust analysis of those patients who might benefit most from each treatment. However, our strongly positive results for the primary outcome, and given the current interest in stratified medicine, provide an indication of those persons with CWP who may be most likely to benefit. This may be helpful given that CBT is not available everywhere in the UK or elsewhere. Finally, we did not restrict the usual care provided by the GP. However, no participant in the TAU arm reported receiving ‘talking therapy’ or exercise therapy at follow-up. With no pharmacological therapies licensed in the UK for fibromyalgia (of which CWP is the cardinal feature), management is likely to have focused on advice, investigation and management of specific reported symptoms.

Reviews of CBT have been conducted in patients with fibromyalgia, however, their conclusions are not completely consistent. Cochrane reviews agree that CBT affects mood and pain positively.^11^ ^31^ While our study reported positive effects in the tCBT arm across all primary and secondary measures 2 years after the end of treatment, with statistically significant improvements in passive coping, kinesiophobia, distress and SF-36 social function subscale (compared to TAU) at p<0.05, none met the more stringent statistical significance cut-off after correction for multiple testing. Most of the evidence to date on the effectiveness of exercise relates to fibromyalgia. Our study extends this evidence of benefit to persons with CWP and it provides evidence that the benefit is long-lasting. It has previously been shown that the effects of an exercise programme on psychological outcomes are maintained long after such a programme has finished and that long-term improvements in patients with fibromyalgia due to increased physical activity are maintained regardless of whether activity levels return to pretreatment levels after active treatment has finished.^32^ It has been demonstrated in a recent meta-analysis that community-deliverable exercise programmes are effective for pain and physical function in adults with osteoarthritis, rheumatoid arthritis and fibromyalgia.^33^ In our study, the only statistically significant difference between exercise and TAU at 2 years after treatment, other than in patient perception of change in their condition, was in the secondary measure SF-36 role emotional, an effect that did not persist after correction for multiple testing. Several guidelines on the management of fibromyalgia recommend the use of multimodal therapy.^34^ ^35^ We therefore hypothesised that the benefits of receiving exercise and tCBT for CWP would be greater than either therapy alone. However, at each follow-up, the effects on the primary outcome measure of the combined therapy were very similar to each intervention delivered alone. Nevertheless, it is noteworthy that, compared with TAU, the most statistically significant differences for secondary outcome measures occurred in the combined treatment group.

In summary, our study has demonstrated for the first time that a short course of either tCBT or exercise for persons with CWP can result in long-term improvements in patients’ global assessment of their condition, compared with TAU. There does not appear to be substantial advantage from providing both interventions. Our work has identified features of patients who may be more likely to respond to tCBT. Finally tCBT has been shown not only to be effective but also highly cost-effective. Future research should focus on: the mechanism by which these improvements might occur; identification of which patients are likely to derive most benefit from these types of non-pharmacological interventions; and investigate novel ways of delivery to further reduce the cost of provision.
